# High-Sensitive Surface Plasmon Resonance Imaging Biosensor Based on Dual-Wavelength Differential Method

**DOI:** 10.3389/fchem.2021.801355

**Published:** 2021-12-08

**Authors:** Youjun Zeng, Jie Zhou, Wei Sang, Weifu Kong, Junle Qu, Ho-Pui Ho, Kaiming Zhou, Bruce Zhi Gao, Jiajie Chen, Yonghong Shao

**Affiliations:** ^1^ Key Laboratory of Optoelectronic Devices and Systems of Ministry of Education and Guangdong Province, College of Physics and Optoelectronics Engineering, Shenzhen University, Shenzhen, China; ^2^ Department of Biomedical Engineering, The Chinese University of Hong Kong, Shatin, Hong Kong SAR, China; ^3^ Aston Institute of Photonic Technologies, Aston University, Birmingham, United Kingdom; ^4^ Department of Bioengineering and COMSET, Clemson University, Clemson, SC, United States

**Keywords:** surface plasmon, biosensing and bioimaging, surface plasmon sensors, biophotonics and plasmonics, biomolecule interaction

## Abstract

Intensity interrogation surface plasmon resonance (ISPR) sensing has a simple schematic design and is the most widely used surface plasmon resonance technology at present. However, it has relatively low sensitivity, especially for ISPR imaging (ISPRi). In this paper, a new technique for the real-time monitoring of biomolecule binding on sensor surfaces *via* ISPRi detection is described. The technique is based on the interrogation of the differential value of two intensities at two specific wavelengths from the reflected light spectrum. In addition, we also optimized the selection of dual-wavelength parameters under different circumstances to achieve the highest sensitivity. The new technique achieved a refractive index resolution (RIR) of 2.24 × 10^–6^ RIU, which is far beyond that of traditional ISPRi technique. Moreover, our new ISPRi technique also realized the real-time detection of high-throughput biomolecular binding. This study is expected to promote the development of faster and more accurate SPRi technologies.

## Introduction

Surface plasmon resonance (SPR) has become a powerful tool for exploring biomolecular interactions in the last decades (Homola, 2008; Schasfoort et al., 2018; Bockova et al., 2019). Four practical interrogation SPR sensing techniques, namely, intensity, wavelength, angle, and phase interrogation SPR modes, have been widely reported (Homola et al., 1999; Liu et al., 2005; Huang et al., 2012). In the wavelength interrogation SPR mode, the incident angle is fixed, and the SPR spectral profile and resonance wavelength can be obtained by scanning the incidence wavelength or using a spectrometer for analyzing the reflected beam. Angular interrogation SPR usually utilizes monochromatic light as excitation light, and the shift of the SPR dip can be monitored through the continuous scanning of the SPR angular spectrograph. In the phase interrogation SPR mode, the SPR phase shift can be obtained by detecting the phase difference between the signal beam and reference beam.

In intensity interrogation SPR (ISPR) mode, the incidence wavelength and angle are usually fixed, and the reflected light intensity is directly monitored to translate the refractive index variation of the sample. This technology avoids the usage of scanning or modulator devices, such as wavelength scanning devices, angle scanning devices, and phase modulators. Compared with the other three interrogation modes, ISPR mode has a simple structure design, easy operational procedure, and cost-effectiveness (Zeng et al., 2017). Moreover, the detecting speed of the ISPR system solely depends on the speed of the light intensity detector, therefore, it has great application potential in 2D real-time biochemical reaction monitoring. In addition, in terms of producibility, ISPR technology, such as Biacore from GE Healthcare and SPR chips from GWC Technologies, is also the most commonly used method in commercial SPR instruments (Nilvebrant, 2018). These instruments use ISPR for DNA hybridization analysis (Mudgal et al., 2020), protein interaction analysis (Nilvebrant, 2018), and cellular analysis (Zhou X. L. et al., 2020).

In addition, sensitivity is a vital parameter in biochemical detection, Researchers have developed many techniques to increase the sensitivity for detecting biomolecular interactions at lower concentrations with lesser sample consumption. And for a typical SPR sensor, a smaller refractive index resolution (RIR) gives a higher system sensitivity. The RIR of a typical ISPR at present is $10^{-5}$ RIU (Homola et al., 1999; Yuk et al., 2005; Zeng et al., 2017), which is 1–2 orders lower compared with those of the other three SPR interrogation modes. The low sensitivity has greatly limited the applications of ISPR in biosensing. In addition, detection throughput is another important parameter for biosensors. By combining imaging technology, multichannel SPR and SPR imaging (SPRi) (Wong and Olivo, 2014; Puiu and Bala, 2016; Zeng et al., 2019; Zhou J. et al., 2020) sensors have been developed to achieve the simultaneous detection of multiple samples at high throughput. However, these multipoint detection schemes are more vulnerable to external noise, and their sensitivities are still limited (Zeng et al., 2019). Zybin et al. proposed a double-wavelength SPR interrogation methods to achieve high-throughput monitoring and high sensitivity; in their system, differential measurements are provided by two diode-lasers, and 2D sensor arrays are used as the detector. The system simultaneously monitored four channels and achieved a refractive index resolution of $5\times 10^{-6}$ RIU (Zybin et al., 2005). However, the two lasers of their system need to be strictly aligned to a common light path to ensure that they reach the same site on the detector after passing through the sensing module. Moreover, the speckles induced by two coherent light sources also caused much noise, especially for intensity-based SPR sensing. Therefore, in this paper, we present a novel ISPRi biosensor based on the differential of light intensities from two selected wavelengths. The system adopts incoherent light with broadband spectrum for excitation, and the intensity information of two selected wavelengths in the reflected light is recorded by two complementary metal oxide semiconductors (CMOSs). We improved the sensitivity of the traditional ISPRi system by about one order and achieved multichannel high-throughput detection. This technique has great potential to be employed in highly sensitive and real-time SPR imaging benchtop devices for molecular interactions detection.

### Principle of the Dual-Wavelength Differential Method

For a Kretschmann-based SPR scheme with gold film (48 nm thickness) on the prism made by SF11, the change in refractive index is from 1.333 RIU to 1.333019 RIU in $10^{-6}$ RIU by volume, the incidence wavelength is from 600 to 700 nm, and the incidence is 54.2°, which is the optimum for 1.333 RIU. The reflection of incidence light obeys the Fresnel Eq. 1, in which ${\text{I}}_{0}\left(\text{λ}\right)$ is the reflective intensity with incidence wavelength λ; $r_{01}$ and $r_{12}$ are the reflection coefficients of prism–metal and metal–sample interfaces, respectively; $\;k_{x}$ and ω are the wave vector and angular frequency of the wave of p-polarized light in incident light; ε is the dielectric constant of the sample; d is the metal thickness, and c is the speed of light in vacuum.

I0(λ)=|r01+r12exp(2iεdε(ωc)2−kx2)1+r01r12exp(2iεdε(ωc)2−kx2)|2 (1)

**FIGURE 1:**
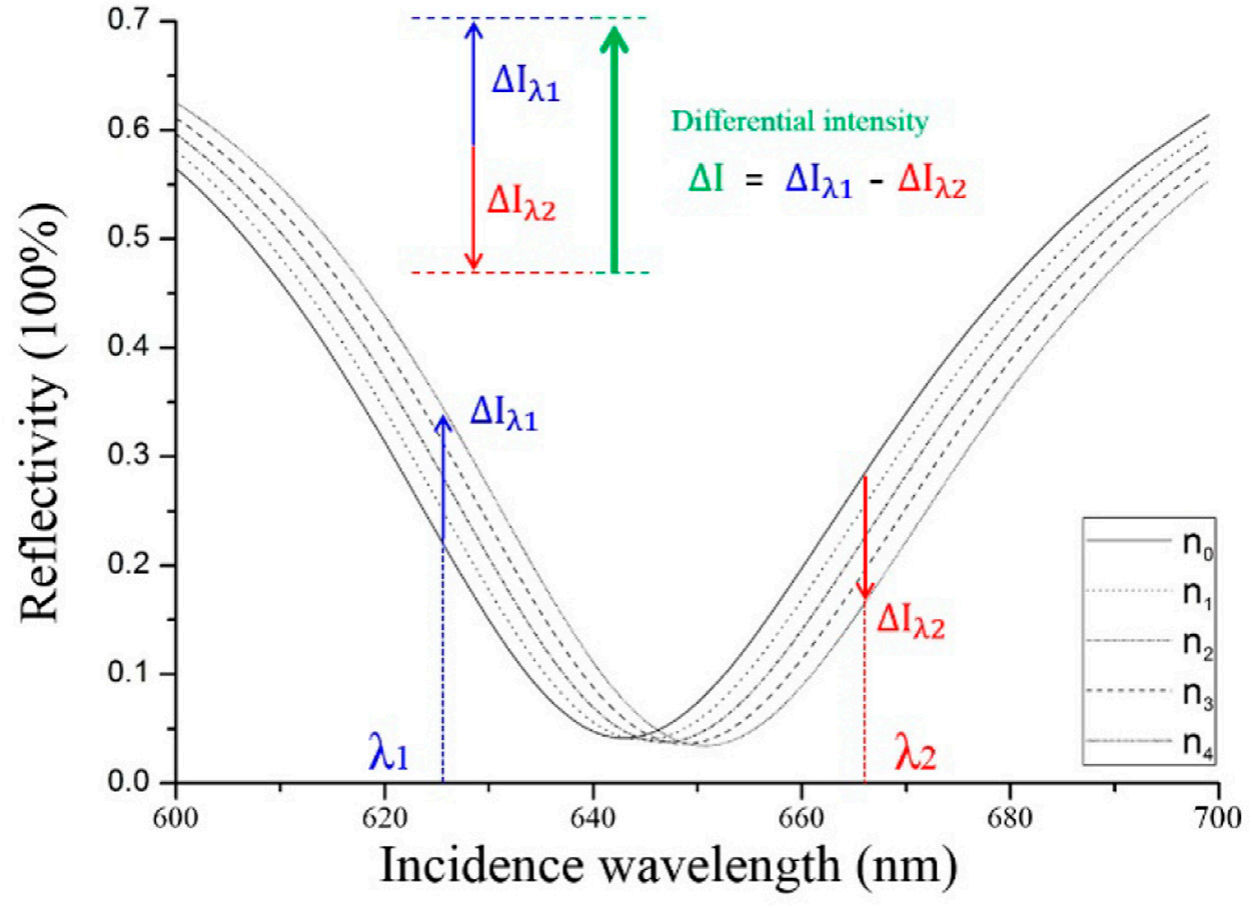
Simulated SPR spectral curves of different sample refractive indexes. $n_{i}$ is a set of linearly increased refractive indexes, *i* = 0, 1, 2, 3, 4, 5.

As it’s illustrated in Figure 1, we simulated the SPR spectral curve under different incident wavelengths. The SPR spectral curve shows two linear regions, in which the reflected light intensity varies linearly with the refractive index. When the refractive index increases, the reflected light intensity increases in one linear region (blue arrow) and decreases in another linear region (red arrow). We can monitor the intensities in the two linear regions at the same time, and make a subtraction ΔI between the two groups of intensity ( ${\text{I}}_{\text{λ}1}-{\text{I}}_{\text{λ}2}$ ), so the intensity change induced by the sample refractive index variation can be amplified.

**FIGURE 2:**
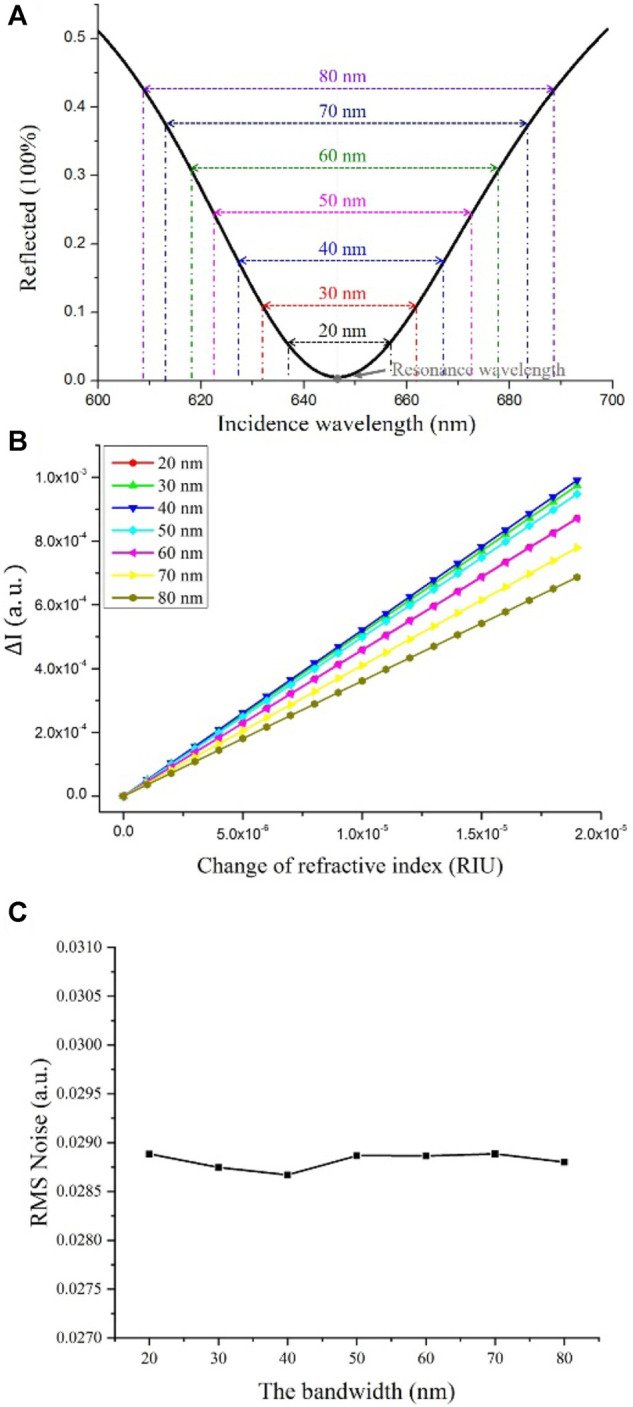
Simulation of intensity amplification optimization and influence of light source noise study. **(A)** Schematic diagram of dual-wavelength selection; **(B)** Dual-wavelength differential value ΔI change with sample refractive index variation. **(C)** The RMS noise of ΔI in different bandwidths.

**FIGURE 3:**
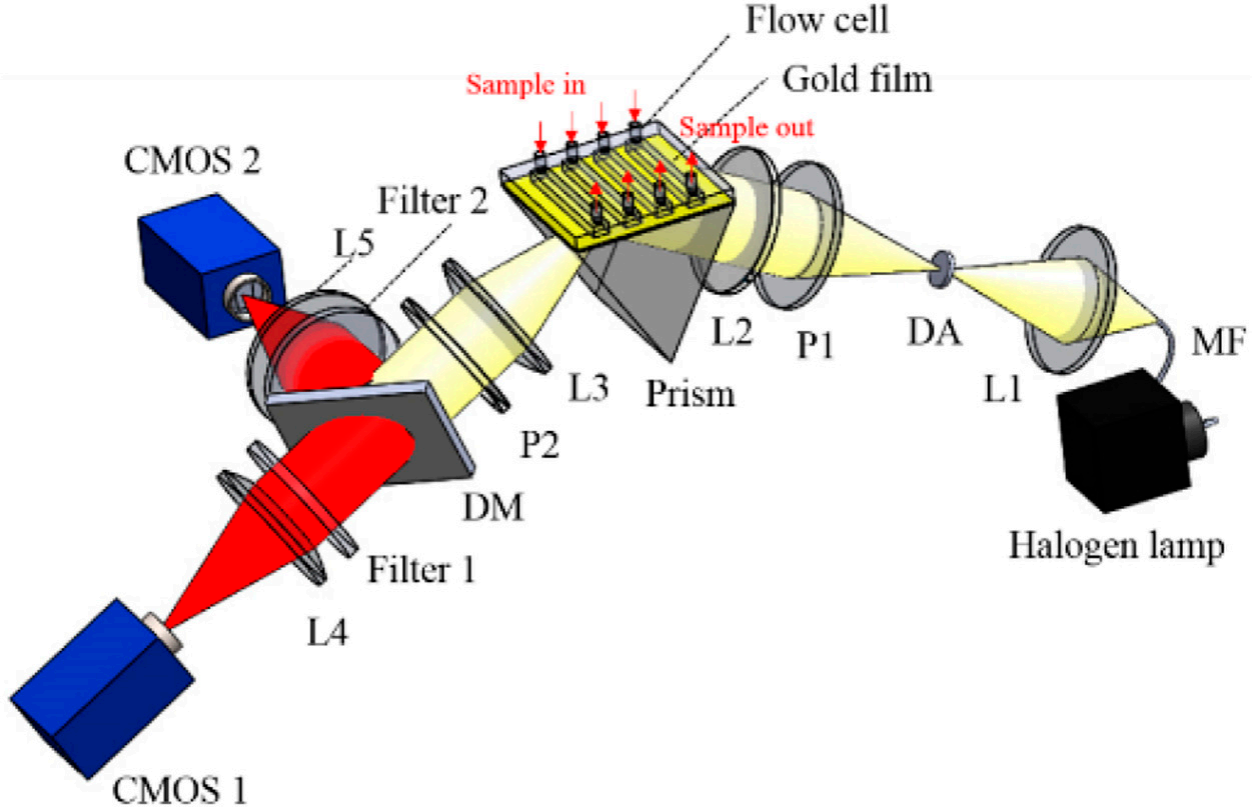
Schematic of ISPRi system based on Kreschmann configuration. L1–L5, lenses; DA, diaphragm aperture; MF, multimode fiber; DM, dichroic mirror; P1 and P2, polarizers.

In addition, dual-wavelength selection needs to be optimized to obtain the highest sensitivity amplification. Based on the simulation results of intensity difference ΔI at different refractive index change. We studied the influence of the bandwidth between the two selected wavelengths to ΔI . As shown in Figure 2A, from the SPR curve, we took the resonance wavelength as the center, symmetrically chose wavelengths of ${\text{λ}}_{1}$ and ${\text{λ}}_{2}$ with different bandwidths, and detected the corresponding reflected light intensities, ${\text{I}}_{\text{λ}1}$ and ${\text{I}}_{\text{λ}2},$ respectively. The relationship between ΔI and the refractive index change in different bandwidth is represented in Figure 2B. The results show that ΔI gives the largest value at the selected bandwidth of 40 nm (dark blue line). Thus, in our experiments, the bandwidth of the dual wavelengths was set to 40 nm, and they were symmetric with respect to the resonance wavelength. Moreover, we also conducted simulation regarding the influence of light source noise. In the simulation process, we added random generated noise $I_{Noise}$ ranging from 0 to 0.01 (identical to the real experiments) to ΔI , then the SPR signal can be expressed as $\Delta I+I_{Noise}$ . After testing of 1000 times, the measured root mean square (RMS) noise obtained under different bandwidth is shown in the Figure 3 (c). It indicates that the RMS noise between different bandwidth is basically identical trivial and the RMS noise variation is within 1%. Therefore, the influence of the noise of the light source can be ignored in our experiments.

### Setup

Figure 3 shows the optical path of the system. A 100 W halogen lamp (GCI-060101, Daheng Optics, Beijing, China) was used as the excitation light source. Divergent light from multimode fiber passes through the collimating lens groups of L1 and L2, and diaphragm aperture to produce parallel lights, then enters into the sensing module. And the sensing module includes prism, sensor chip, and flow cell. The parallel light is coupled into the prism to excite the SPR effect on the sensor chip in the gold film.

After the reflected light passes through L3 and P2, it is split by a 635 nm low-pass dichroic mirror (FF635-Di01- 25×36 , Semrock, New York, United States). And two laser line filters of filter 1 (7671, Alluxa, Santa Rosa, United States) and filter 2 (7261, Alluxa, Santa Rosa, United States) are used to filter out the two operating wavelengths. Their center wavelengths are 608 and 650 nm, and their full width at half maximum is 1 nm. The system is controlled by a homemade LabVIEW program. During the test, two CMOSs acquire the images of the sensing surface, simultaneously. The control program differentiates the two intensities at two different wavelengths in real time.

## Results and Discussions

**FIGURE 4:**
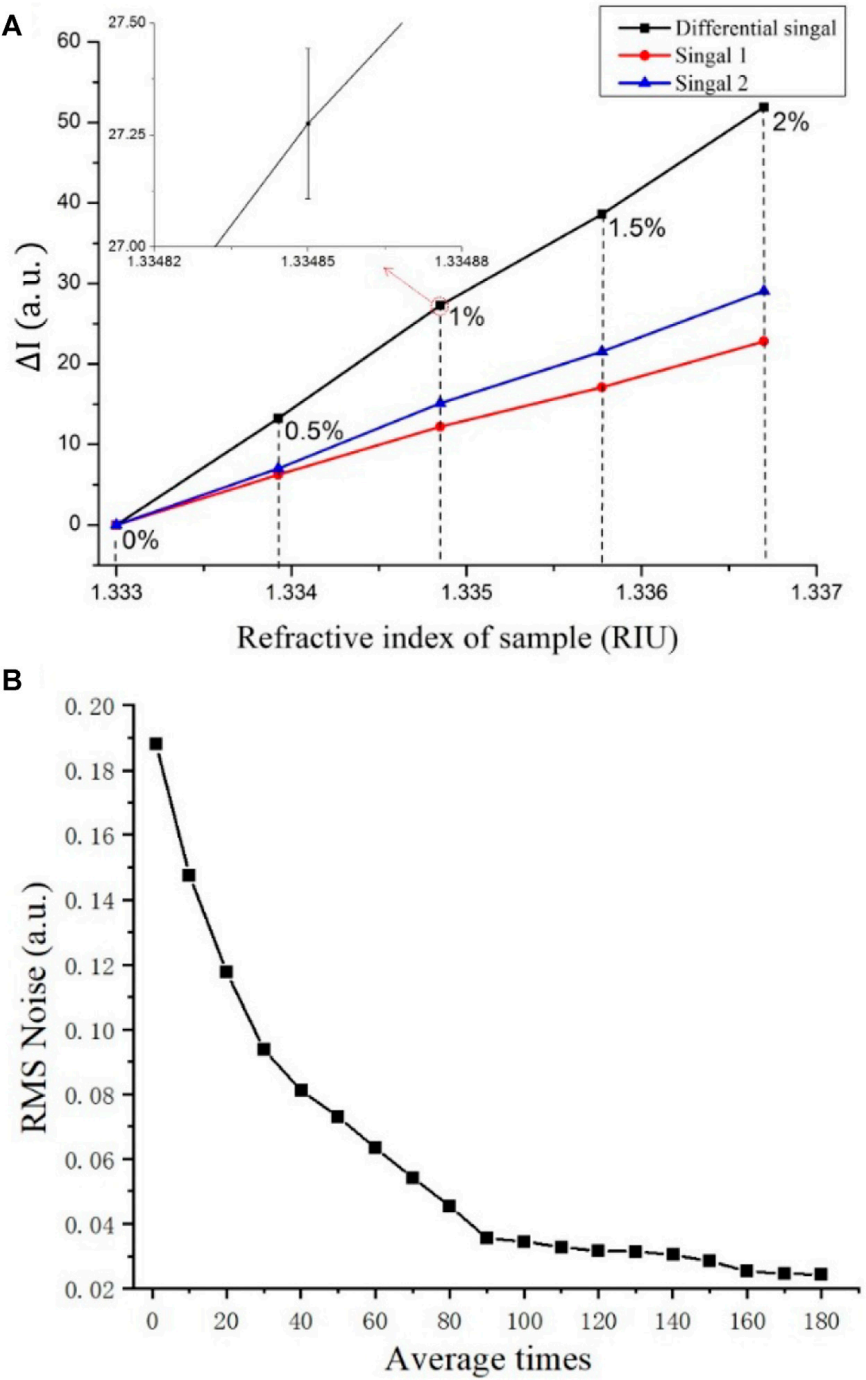
Sensitivity test results. **(A)** Resonance wavelength shift obtained from low-concentration saline water; **(B)** Relation between averaging number and RMS noise.

We detected the intensity shift induced by small variations in saline water concentration to determine the refractive index resolution of our system. We tested a series of saline water with concentrations of 0–2% in increments of 0.5% by volume, which correspond to the refractive indexes ranging from 1.3330 RIU to 1.3367 RIU. During the test, the incident angle was fixed to 54.9°, which corresponds to the resonance wavelength of 630 nm and sample’s refractive index of 1.3330 RIU. Before the test, the system obtained the image of the whole sensor surface, and we evenly divided the entire sensing surface into 3 × 3 array sensor sites. And the nine sites were monitored simultaneously. The results are illustrated in Figure 4A. $I_{\text{λ}1}\;$ and $I_{\text{λ}2}$ are the changes in intensity that were collected simultaneously by CMOS 1 and CMOS 2, and the black curve is the ΔI obtained after the differential of $I_{\text{λ}1}$ and $I_{\text{λ}2}$ . Note that each data point in the figure is the average value of nine detection sites. The inset is the zoom-in figure at 1% saline water concentration.

We also detected the noise of our ISPRi system. Noise is reduced by averaging multiple acquisitions. The relationship between average times and RMS noise is shown in Figure 4B. RMS noise was reduced with the increase in averaging number and tended to be stable and small when the averaging number exceeded 100 times. In our experiments, averaging number was set to 100 to ensure best performance. The frame rates of the two CMOSs were 100 fps, so the imaging time for acquiring one SPR image is 1 s. Therefore, the real-time refractive index monitoring ability of our SPR system is verified.

And the refractive index resolution (RIR) of the system can be calculated from Eq. 2 (Zeng et al., 2020a; Ng et al., 2013)

$$\sigma_{RI}=\frac{\delta n}{\delta s}\text{Δ}\sigma_{SD}$$ (2)

where $\sigma_{RI}$ is the RIR, δn is the refractive index change, δs is the change in intensity, and $\text{Δ}\sigma_{SD}$ is the RMS noise of our ISPRi system. The samples with concentrations of 0.5–1% caused the maximum intensity change. In this case, the values of δn , δs , and $\text{Δ}\sigma_{SD}$ were 0.000925 RIU, 14.04 a.u., and 0.034 a.u., respectively. From the above calculation, we obtained the RIR of $2.24\times 10^{-6}$ RIU.

In addition, we also tested the biosensing capability of our ISPRi sensor in detecting the interactions between two kinds of protein molecules. The biomolecule binding events on the sensing surface is indicated by the change in refractive light intensity (Mudgal et al., 2020). The sensor chip was rinsed with deionized water and dried with nitrogen before being placed on the prism for the biological experiment. The chip was attached to the coupling prism using a small drop of refractive index matching oil. And a four-channel PDMS flow chamber was adopted for sample injection.

For the pretreatment of the sensing surface, the antigen of human IgG was bound to the gold film *via* physical absorption process and real-time monitoring was performed (Zeng et al., 2020b; Miyan et al., 2021; Zhou et al., 2022). First, phosphate-buffered saline (PBS, 0.01 M, pH 7.4) was separately injected into the four channels and flushed for 5 min. Second, human IgG (100 μg/ml) was injected into each channel simultaneously in a flow rate of 10 μL/min until the image intensities were stabilized. Then, PBS was injected and flushing. After the images are stable, bovine serum albumin solution in the concentration of 10 mg/ml was injected into the four channels for 5 min to occupy the possible vacancy sites without the binding of human IgG. Therefore, the nonspecific binding of goat anti-human IgG to the surface in the following procedure can be prevented. Then, the PBS solution are injected to each channel for 10 min so as to obtain the baseline for the following bio-experiments.

In the biological binding process. The four channels were injected with PBS buffer and goat anti-human IgG in concentrations of 1, 2, and 5 μg/ml respectively. The channel with PBS was used as the reference channel, and the three other channels were the reaction channels. The differential intensities of all channels were recorded. After the intensity became stationary, PBS was injected to wash the nonspecific binding of goat anti-human IgG to the sensing surface.

**FIGURE 5:**
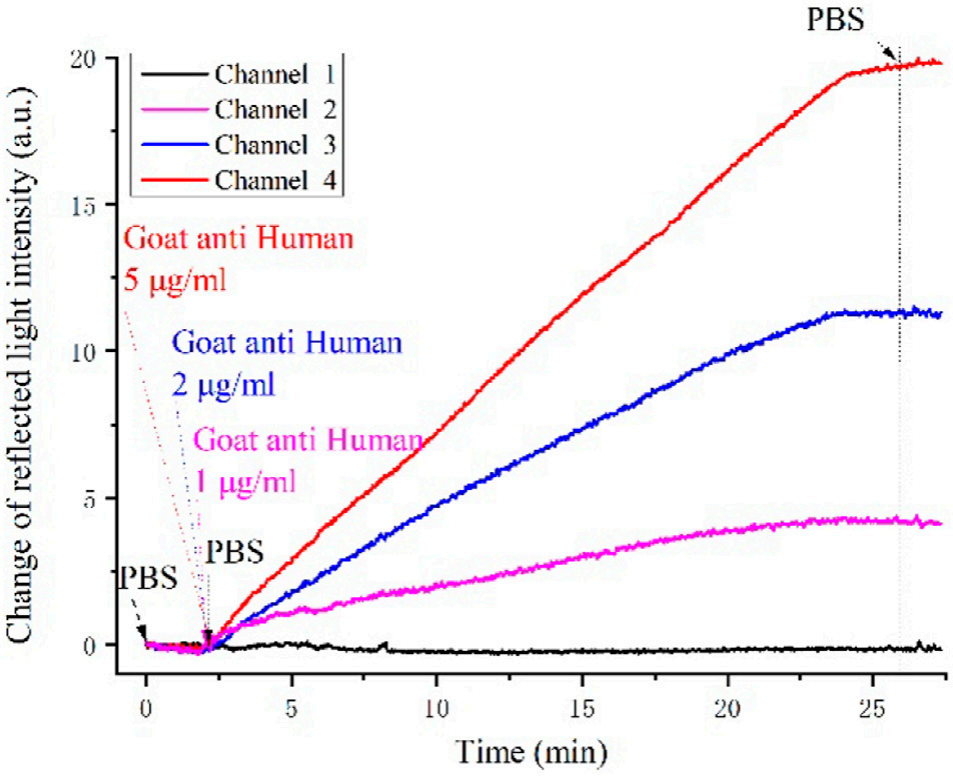
Real-time intensity variation in the antigen-antibody interaction between goat anti-human IgG and human IgG.

The real-time monitoring reflected the light intensities of the four channels as shown in Figure 5. The black curve is the reference channel, and the curves with different colors are the reaction channels. The ΔI values of the three reaction channels increased linearly with the sample concentration in the biochemical binding process, while the reference channel did not obviously change.

**FIGURE 6:**
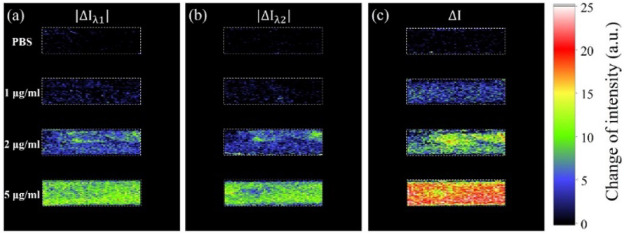
SPR Intensity changes of the four channels treated with PBS and 1, 2, and 5 μg/ml human IgG. **(A,B)** are the images of the intensity based on single wavelength of $\;{\text{λ}}_{1}$ and ${\text{λ}}_{2}$ . And **(C)** is the image of ΔI .

Another unique advantage of our system is its SPR imaging ability. The SPR image of the entire sensing surface can be obtained simultaneously during the detection. As illustrated in Figure 6, the color of each pixel represents the intensity shift, which indicates the biomolecule binding situation on the corresponding sensing site after the reaction. The light intensity is shown in the false color map. Figures 6A,B are the images of the single-wavelength intensity shifts of ${\text{λ}}_{1}$ and ${\text{λ}}_{2}$ , which were directly captured by CMOS 1 and CMOS 2, respectively. Figure 6C is total intensity shift induced by the intensity variation of the two wavelengths. Therefore, this high-sensitivity ISPRi scheme gives a higher response compared with single-wavelength SPR sensing. Moreover, the proposed method is also favorable for real-time 2D bio-reaction monitoring, which means that one can monitor the whole 2D sensing sites in real time and select the region of interest anytime during the test.

## Conclusion

In summary, we developed a versatile real-time ISPRi system, which has improved sensitivity based on the differential light intensities from two selected wavelengths. This technique has several advantages compared with traditional ISPR sensors. The proposed technique achieved a higher sensitivity and higher detection throughput in a low-cost optical configuration without using any mechanical moving parts or optical scanning devices compared with other sensors. The results have shown that the SPR refractive index resolution of the scheme is $2.24\times 10^{-6}$ RIU, which produces the highest sensitivity in the existing ISPRi system so far. The ability of the developed sensor in the real-time detection of multiple bio-samples was also verified. Nevertheless, the system’s sensitivity still has much room for improvement. For example, the differential intensity can be further enlarged by replacing the dual wavelengths from the visible region to the near-infrared region, and the noise can be reduced by employing a high-brightness light source for SPR excitation. Moreover, in terms of SPR imaging, the high-performance 2D detector can be used to suppress dark noise. The imaging resolution and field of view can also be improved if imaging lens groups with high performance are adopted in the future.

## Data Availability

The raw data supporting the conclusions of this article will be made available by the authors, without undue reservation.
