# Corrigendum: High-Sensitive Surface Plasmon Resonance Imaging Biosensor Based on Dual-Wavelength Differential Method

**DOI:** 10.3389/fchem.2022.849460

**Published:** 2022-02-24

**Authors:** Youjun Zeng, Jie Zhou, Wei Sang, Weifu Kong, Junle Qu, Ho-Pui Ho, Kaiming Zhou, Bruce Zhi Gao, Jiajie Chen, Yonghong Shao

**Affiliations:** ^1^ Key Laboratory of Optoelectronic Devices and Systems of Ministry of Education and Guangdong Province, College of Physics and Optoelectronics Engineering, Shenzhen University, Shenzhen, China; ^2^ Department of Biomedical Engineering, The Chinese University of Hong Kong, Hong Kong SAR, China; ^3^ Aston Institute of Photonic Technologies, Aston University, Birmingham, United Kingdom; ^4^ Department of Bioengineering and COMSET, Clemson University, Clemson, SC, United States

**Keywords:** surface plasmon, biosensing and bioimaging, surface plasmon sensors, biophotonics and plasmonics, biomolecule interaction

In the original article there were errors in the Affiliation section. Affiliations 3 and 4 need to be in reversed order. Affiliation 3 should be "Aston Institute of Photonic Technologies, Aston University, Birmingham, United Kingdom". Affiliation 4 should be "Department of Bioengineering and COMSET, Clemson University, Clemson, SC, United States". Affiliation 1 is also corrected to "Key Laboratory of Optoelectronic Devices and Systems of Ministry of Education and Guangdong Province, College of Physics and Optoelectronics Engineering, Shenzhen University, Shenzhen, China".

The authors apologize for this error and state that this does not change the scientific conclusions of the article in any way. The original article has been updated.

